# Vertebrate TFPI-2 C-terminal peptides exert therapeutic applications against Gram-negative infections

**DOI:** 10.1186/s12866-016-0750-3

**Published:** 2016-06-27

**Authors:** Gopinath Kasetty, Emanuel Smeds, Emelie Holmberg, Louise Wrange, Selvi Adikesavan, Praveen Papareddy

**Affiliations:** Division of Respiratory Medicine and Allergology, Lund University, Lund, Sweden; Division of Infection Medicine, Department of Clinical Sciences, Lund University, Biomedical Center, B14, Tornavägen 10, SE-221 84 Lund, Sweden; School of Biosciences and Technology, Environmental Division, VIT University, Tamil Nadu, India; Dept of Biotechnology, DKM college for Women, Sainathapuram, 632001 Vellore, India

**Keywords:** TFPI-2, Peptide, Complement, Vertebrates, Antimicrobial, Coagulation, Sepsis, Evolution

## Abstract

**Background:**

Tissue factor pathway inhibitor-2 (TFPI-2) is a serine protease inhibitor that exerts multiple physiological and patho-physiological activities involving the modulation of coagulation, angiogenesis, tumor invasion, and apoptosis. In previous studies we reported a novel role of human TFPI-2 in innate immunity by serving as a precursor for host defense peptides. Here we employed a number of TFPI-2 derived peptides from different vertebrate species and found that their antibacterial activity is evolutionary conserved although the amino acid sequence is not well conserved. We further studied the theraputic potential of one selected TFPI-2 derived peptide (mouse) in a murine sepsis model.

**Results:**

Hydrophobicity and net charge of many peptides play a important role in their host defence to invading bacterial pathogens. In vertebrates, the C-terminal portion of TFPI-2 consists of a highly conserved cluster of positively charged amino acids which may point to an antimicrobial activity. Thus a number of selected C-terminal TFPI-2 derived peptides from different species were synthesized and it was found that all of them exert antimicrobial activity against *E. coli* and *P. aeruginosa*. The peptide-mediated killing of *E. coli* was enhanced in human plasma, suggesting an involvement of the classical pathway of the complement. Under in vitro conditions the peptides displayed anti-coagulant activity by modulating the intrinsic pathway of coagulation and in vivo treatment with the mouse derived VKG24 peptide protects mice from an otherwise lethal LPS shock model.

**Conclusions:**

Our results suggest that the evolutionary conserved C-terminal part of TFPI-2 is an interesting agent for the development of novel antimicrobial therapies.

**Electronic supplementary material:**

The online version of this article (doi:10.1186/s12866-016-0750-3) contains supplementary material, which is available to authorized users.

## Background

In response to pathogenic microorganisms, host organisms have evolved a diverse range of defense mechanisms, starting from simple mechanical barriers to complex immune systems. In these processes, blood coagulation, complement cascades and antimicrobial peptides are central to host defense and many components of these systems are evolutionary conserved [1]. The crosstalk between the coagulation and complement systems is essential for the clearance of the infection. Together they have a dual role of immobilization and destruction of invading bacteria in addition to preventing the loss of body fluids. Coagulation factors, apart from their primary role of maintaining hemostasis, are also involved in killing of bacteria and immunomodulation, such as thrombin [2], kininogen [3], protein C inhibitor [4], fibrinogen [5, 6], antithrombin [7], TFPI-1 [8], and TFPI-2 [9, 10]. These proteins may explore their activities either as intact molecules or after proteolysis. Often they execute their host defense functions by killing the intruder or by triggering immunomodulatory reactions. Many well-characterized antimicrobial peptides have recently been found to exhibit also multifaceted immunomodulatory activities, such as reported for LL-37 [11, 12] and some antimicrobial peptides have been described to be involved in angiogenesis, chemotaxis, and wound-healing [11]. These biological properties suggest that host defense peptides may have a clinical potential also in disorders where targeting of inflammatory pathways is beneficial, such as in sepsis [11, 13].

Tissue factor pathway inhibitor 2 (TFPI-2) consists of a highly negatively charged N-terminal region, three tandemly linked Kunitz-type domains, and a highly positively charged C-terminus [14, 15]. The molecule is synthesized and secreted by many cells, including skin fibroblasts, endothelial cells (ECs), smooth muscle cells (SMCs), dermal fibroblasts, keratinocytes, monocytes, macrophages and syncytiotrophoblasts [16, 17]. In vitro, TFPI-2 is a weak inhibitor of coagulation induced by the TF-VII complex, while it targets a wide range of proteases such as trypsin, chymotrypsin, plasmin, MMPs, factor XIa and plasma kallikrein [18, 19]. Stimulation of human umbilical vein endothelial cells with inflammatory mediators such as PMA, LPS, or TNF-α significantly increases TFPI-2 expression [17]. Analogously, in a murine model, TFPI-2 expression is dramatically upregulated in the liver upon LPS stimulation [20]. Notably, it has been shown that proteases such as ADAMTS1, plasmin and thrombin can process TFPI-2 at its C-terminal end in in vitro experiments [21]. We previously reported an undisclosed host defense function of the C-terminal region of TFPI-2 [9, 10]. TFPI-2 as well as the C-terminal peptides of the molecule were detected in wounds from patients and were found in complex with the bacteria and fibrin. Correspondingly, human TFPI-2 was degraded in vitro by human neutrophil elastase, leading to the generation of C-terminal TFPI-2 fragments. These fragments were then found to bind to various bacterial surfaces, kill gram-negative bacteria, through membrane lysis, and boost complement activation, including formation of the membrane attack complex (MAC) and antimicrobial C3a. In a therapeutic context, the peptide significantly reduced mortality either as a monotherapy, or in combination with ceftazidime *E. coli* and *P. aeruginosa* sepsis models. We therefore hypothesized that TFPI-2 C-terminal derived peptides may have an important function in the host defense to infection, thus they may be interesting agents for drug development.

## Results

### Phylogenetic and sequence analysis of the TFPI-2 C-terminal part from different species

**Fig. 1:**
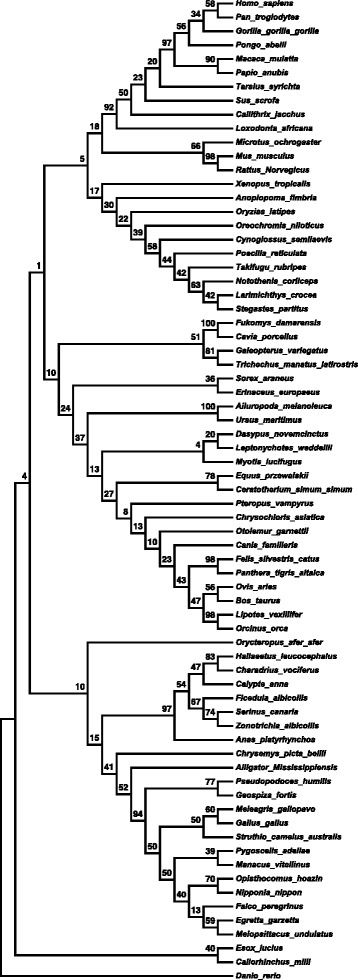
Phylogenetic tree analysis of the C-terminal region of TFPI-2 from vertebrates. Phylogenetic tree from 72 vertebrate TFPI-2 species was constructed using Neighbour-Joining tree with 1000 bootstrap replications on MEGA6

**Fig. 2:**
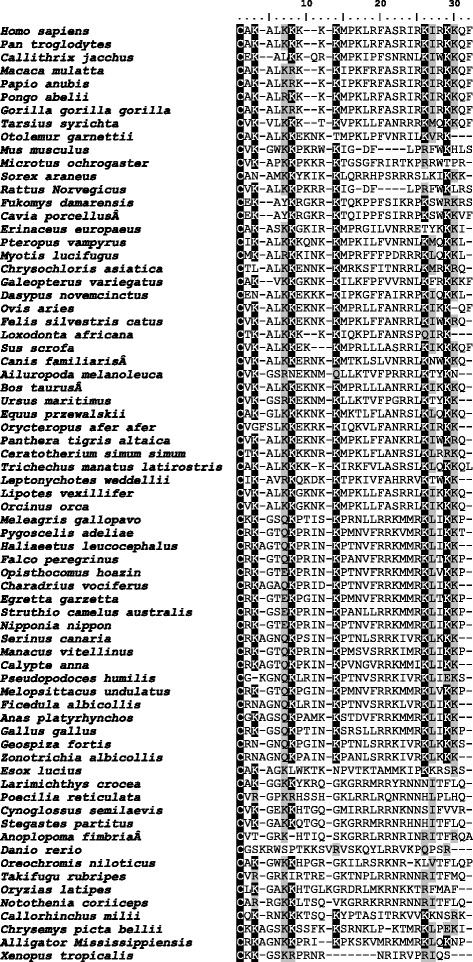
Sequence homology of the C-terminal region of TFPI-2. ClustalW multiple sequence alignment of TFPI-2 where identical and similar amino acids in all sequences are highlighted in black and grey shaded, respectively

A phylogenetic tree was constructed with C-terminal TFPI-2 sequences from 72 vertebrate organisms, using neighbor-joining method with 1000 bootstrap repeats, resulting in distinct groups (Fig. 1). The remainder of TFPI-2 and full length protein are more conserved than the C-terminal region, at first suggesting that the C-terminal region has not been under evolutionary pressure (Additional file 1: Figure S1). Even though the TFPI-2 amino acid length varies among the species, many other conserved regions were observed among the species (data not shown). Interestingly, the multiple sequence alignment of the C-terminal antimicrobial peptide region is not fully conserved (Fig. 2). Importantly however, even though the C-terminal region was not well conserved, the net positive charge is preserved, with a charge ranging from +7 to +14 (Table 1). This suggests an important role of the charge and points towards putative antimicrobial activity where charge rather than the exact amino acid sequence is essential.

**Table 1:** Multiple sequence alignment of vertebrate TFPI-2 C-terminal region showing identical and similar amino acids

Entry & name	Latin & name	English & name	Protein & sequence	Net & charge
		MAMMALS		
P48307	*Homo sapiens*	Human	CAKALKKKKKMPKLRFASRIRKIRKKQF	14
H2QUX8	*Pan troglodytes*	Chimpanzee	CAKALKKKKKMPKLRFASRIRKIRKKQF	14
U3ENJ1	*Callithrix jacchus*	White-tufted-ear marmoset	CEKALKKQRKMPKIPFSNRNLKIWKKQF	9
F7CWN2	*Macaca mulatta*	Rhesus macaque	CAKALKRKKKIPKFRFASRIRKIRKKQF	14
XP_003896360.1	*Papio anubis*	Olive baboon	CAKALKRKKKIPKFRFASRIRKIRKKQF	14
H2PMX0	*Pongo abelii*	Sumatran orangutan	CAKALRKKKKMPKLRFASRIRKIRKKQF	14
G3QPC8	*Gorilla gorilla gorilla*	Western lowland gorilla	CAKALKRKKKMPKLRFASRIRKIRKKQF	14
XP_008067041.1	*Tarsius syrichta*	Philippine tarsier	CVKVLKKKTKVPKLLFANRRRKMQKKQF	12
H0XT57	*Otolemur garnettii*	Small-eared galago	CAKALKKEKNKTMPKLPFVNRILKVRK	9
O35536	*Mus musculus*	Mouse	CVKGWKKPKRWKIGDFLPRFWKHLS	7
XP_005363735.1	*Microtus ochrogaster*	Prairie Vole	CVKAPKKPKKRKTGSGFRIRTKPRRWTP	12
XP_004602345.1	*Sorex araneus*	European shrew	CANAMKKYKIKKLQRRHPSRRRSLKIKK	13
EDL84392.1	*Rattus Norvegicus*	Brown Rat	CVKALKKPKRRKIGDFLPRFWKLRS	9
XP_010601797.1	*Fukomys damarensis*	Damaraland Mole Rat	CEKAYKRGKRKTQKPPFSIKRPKSWRKR	12
H0VU83	*Cavia porcellus*	Guinea pig	CEKAYKRGKRKTQIPPFSIRRPKSWKKV	10
XP_007522577.1	*Erinaceus europaeus*	Western European Hedgehog	CAKASKKGKIRKMPRGILVNRRETYKKK	11
XP_011361893.1	*Pteropus vampyrus*	Large Flying Fox	CIKALKKKQNKKMPKILFVNRNLKMQKK	11
G1PX03	*Myotis lucifugus*	Little brown bat	CMKALRKKINKKMPRFFFPDRRRKLQKK	12
XP_006834353.1	*Chrysochloris asiatica*	Cape Golden Mole	CTLALKKENNKKMRKSFITNRRLKMRKR	11
XP_008569808.1	*Galeopterus variegatus*	Sunda flying lemur	CAKVKKGKNKKILKFPFVVRNLKFRKKK	13
XP_004475751.1	*Dasypus novemcinctus*	Nine-banded Armadillo	CENALKKEKKKKIPKGFFAIRRPKIQKK	10
W5NS10	*Ovis aries*	Sheep	CVKALKKEKNKKMPRLLFANRRLKIKKQ	11
M3X7K4	*Felis silvestris catus*	Cat	CVKALKKEKNKKMPKLFFANRRLKIWKR	11
G3THG9	*Loxodonta africana*	African elephant	CTKALKKKKKIQKPLFANRSPQIRK	10
F1SFC1	*Sus scrofa*	Pig	CVKALKKEKKMPRLLLASRRLKIKKKQF	11
E2RBF0	*Canis familiaris*	Dog	CVKALKKERNKKMTKLSLVNRRLKNWKK	11
G1MHA9	*Ailuropoda melanoleuca*	Giant panda	CVKGSRNEKNMQLLKTVFPRRRLKTYKN	8
Q7YRQ8	*Bos taurus*	Bovine	CVKALKKEKNKKMPRLLLANRRLKIKKK	12
XP_008684473.1	*Ursus maritimus*	Polar bear	CVKGSRKEKNMKLLKTVFPGRRLKTYKK	10
XP_008542988.1	*Equus przewalskii*	Przewalski’s horse	CAKGLKKKKNKKMKTLFLANRSLKLQKK	12
XP_007938042.1	*Orycteropus afer afer*	Aardvark	CVGFSLKKEKRKKIQKVLFANRRLKIRK	11
XP_007076762.1	*Panthera tigris altaica*	Amur Tiger	CVKALKKEKNKKMPKLFFANKRLKIWKR	11
XP_004431409.1	*Ceratotherium simum simum*	Southern White Rhinoceros	CTKALKKKKNRKMPKLFLANRSLKLRRK	13
XP_004386213.1	*Trichechus manatus latirostris*	Florida manatee	CAKALKKKKKKIRKFVLASRSLKLQKKQ	13
XP_006733001.1	*Leptonychotes weddellii*	Weddell seal	CIKAVRKQKDKKTPKIVFAHRRVKTWKK	11
XP_007450700.1	*Lipotes vexillifer*	Yangtze River dolphin	CVKALKKGKNKKMPKLLFASRRLKIKKK	13
XP_004265641.1	*Orcinus orca*	Killer Whale	CVKALKKGKNKKMPKLLFASRRLKIKKK	13
		BIRDS		
XP_010710984	*Meleagris gallopavo*	Wild Turkey	CKKGSQKPTISKPRNLLRRKMMRKLIKK	12
XP_009328855.1	*Pygoscelis adeliae*	Adélie penguin	CRKGTQKPRINKPMNVFRRKVMRKLIKK	12
XP_010560815	*Haliaeetus leucocephalus*	Bald eagle	CRKAGTQKPRINKPTNVFRRKMMRKLIK	11
XP_005228880.1	*Falco peregrinus*	Peregrine falcon	CRKGTQKPRINKPANVFRRKMMRKLTKK	12
XP_009934468.1	*Opisthocomus hoazin*	Hoatzin	CRKGTEKPRINKPTNVFRRKMMRKLVKK	11
XP_009893654.1	*Charadrius vociferus*	Killdeer	CRKAGAQKPRIDKPTNVFRRKMMRKLIK	10
XP_009639692.1	*Egretta garzetta*	Little egret	CRKGTEKPGINKPMNVFRRKMMRKLTKK	10
XP_009672625.1	*Struthio camelus australis*	Ostrich	CRKGSEKPRINKPANLLRRKMMRKLIKK	11
XP_009464906.1	*Nipponia nippon*	Crested Ibis	CRKGTEKPRINKPTNVFRRKMMRKLIKK	11
XP_009084577.1	*Serinus canaria*	Atlantic canary	CRKAGNQKPSINKPTNLSRRKIVRKLKK	11
XP_008919443.1	*Manacus vitellinus*	Golden-collared manakin	CRKGTQKPRINKPMSVSRRKIMRKLIKK	12
XP_008492487.1	*Calypte anna*	Anna’s hummingbird	CRKAGTQKPKINKPVNGVRRKMMIKLIK	11
XP_005518860.1	*Pseudopodoces humilis*	Ground tit	CGKGNQKLRINKPTNVSRRKIVRKLIEK	9
XP_005152979.1	*Melopsittacus undulatus*	Budgerigar	CRKGTQKPGINKPMNVFRRKMMRKLVKK	11
XP_005041216.1	*Ficedula albicollis*	The collared flycatcher	CRNAGNQKLRINKPTNVSRRKLVRKLIK	10
XP_005010988.1	*Anas platyrhynchos*	Mallard	CGKAGSQKPAMKKSTDVFRRKMMRKLIK	9
XP_418662.2	*Gallus gallus*	Chicken	CRKGSQKPTINKSRSLLRRKMMRKLIKK	12
XP_005418689.1	*Geospiza fortis*	Medium ground finch	CRNGNQKPGINKPTNLSRRKIVRKLKKK	11
XP_005480303.1	*Zonotrichia albicollis*	White-throated Sparrow	CRNAGNQKPAINKPANLSRRKIVRKLKK	10
		REPTILES		
XP_005308608.1	*Chrysemys picta bellii*	Western painted turtle	CKKAGSKKSSFKKSRNKLPKTMRKLPEK	11
XP_006258519.1	*Alligator Mississippiensis*	American Alligator	CRKAGNKKPRIKPKSKVMRKMMRKLQKN	13
		AMPHIBIANS		
Q5FVY6	*Xenopus tropicalis*	Western clawed frog	CKKGSKRPRNRNRIRVPRIQS	9
		FISHES		
XP_010889816.1	*Esox lucius*	The northern pike	CAKAGKLWKTKNPVTKTAMMKIPKKRSR	10
XP_010739844.1	*Larimichthys crocea*	Croceine croaker	CAKGGKKYKRQGKGRRMRRYRNNNITFL	11
XP_008419957.1	*Poecilia reticulata*	Guppy	CVRGPKRHSSHGKLRRLRQNRNNHLPLH	8
XP_008329541.1	*Cynoglossus semilaevis*	Cynoglossus	CVKGEKKHTGQGMIRRLRRNKNNSIFVV	7
XP_008287494.1	*Stegastes partitus*	Bicolor damselfish	CVKGAKKQTGQGKGRRMRRNRHNHITFL	9
XP_007259866.1	*Mexican tetra*	Astyanax mexicanus	CARKSKPGKMRRKLIPRKPERRI	10
C3KHI5	*Anoplopoma fimbria*	Sablefish	CVTGRKHTIQSKGRRLRRNRINRITFRQ	10
Q1WCN6	*Danio rerio*	Zebrafish	CGSKRWSPTKKSVRVSKQYLRRVKPQPS	9
I3JXJ8	*Oreochromis niloticus*	Nile tilapia	CAKGWKKHPGRGKILRSRKNRKLVTFLQ	10
XP_003969359.1	*Takifugu rubripes*	Japanese Puffer	CVRGRKIRTREGKTNPLRRNRNNRITFM	9
XP_004073873.1	*Oryzias latipes*	Japanese Rice Fish	CLKGAKKHTGLKGRDRLMKRNKKTRFMA	10
XP_010772945.1	*Notothenia coriiceps*	Black Rock Cod	CARRGKKLTSQVKGRRKRRNRNNRITFL	12
XP_007903512.1	*Callorhinchus milii*	Australian Ghostshark	CQKRNKKKTSQKYPTASITRKVVKKNSR	11

### Antimicrobial activities of TFPI-2 C-terminal derived peptides

**Fig. 3:**
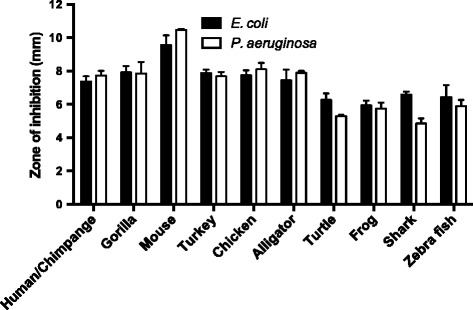
Antimicrobial activities of TFPI-2 C-terminal derived peptides from different species. For determination of antimicrobial activities, *E. coli* ATCC 25922 and *P. aeruginosa* ATCC 27853 (4 × 10^6^ cfu) were inoculated in 0.1 % TSB agarose gel. Each 4 mm-diameter well was loaded with 6 μl of peptide (at 100 μM). The zones of clearance (in mm) correspond to the inhibitory effect of each peptide after incubation at 37 °C for 18–24 h (mean values are presented, *n* = 3). A control with buffer only yielded no inhibition zone

**Fig. 4:**
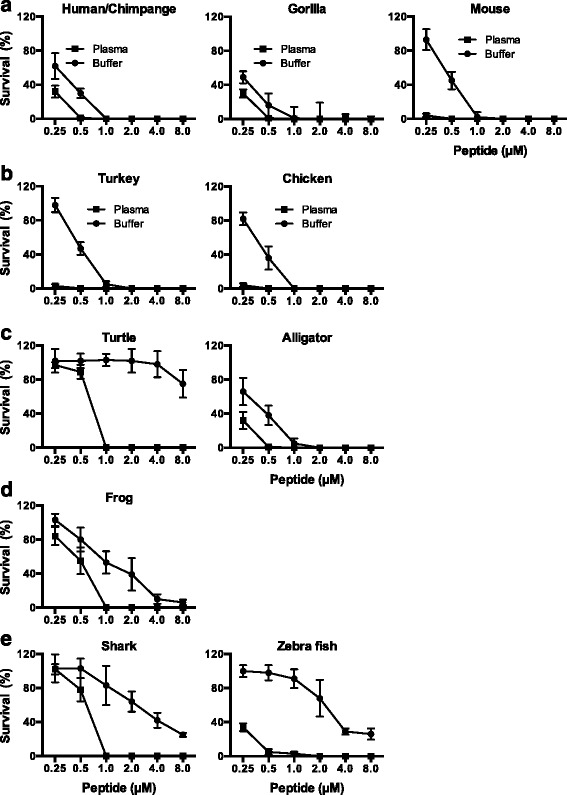
Activities of TFPI-2 C-terminal derived peptides in human plasma. The bactericidal activity of TFPI-2 peptides was assessed in physiological buffer conditions using viable count analysis. **a**–**e** Bacteria *E. coli* ATCC 25922 were grown to mid-logarithmic phase and incubated with varying concentrations of peptides corresponding to sequences found in mammals, birds, reptiles, frog and fish species. Peptides were used in buffer containing 0.15 M NaCl, 10 mM Tris, pH 7.4 alone, or in presence of 20 % human plasma. The antimicrobial activity was determined by plating serial dilution of bacteria on TH agar plates and number of cfu was counted after overnight incubation. All investigated peptides showed dose dependent bactericidal activity and 100 % bacterial killing was achieved in all cases at 1 μM concentration

Having identified the C-terminal region of TFPI-2, we next wished to investigate whether other vertebrate derived peptides retain similar bactericidal activity compared to the human peptide. The antimicrobial properties of the C-terminal TFPI-2 derived peptides from different vertebrates were assessed using radial diffusion assay against gram-negative bacteria *E. coli* and *P. aeruginosa* (Fig. 3). These species included primates (human and gorilla), rodents (mouse), birds (turkey and chicken), reptiles (alligator and turtle), amphibians (frog) and fishes (shark and zebra fish). We also noted that all vertebrate peptides displayed antimicrobial activities at concentrations of 100 μM against both bacteria. In consensus with the human peptide, we investigated if also other vertebrate-derived peptides display enhanced bactericidal activity in presence of plasma. To this end, the peptides were incubated at varying concentrations with *E. coli* in presence of physiological buffer containing 20 % human citrate plasma, since this *E. coli* strain is complement sensitive. In concordance with previous data, all vertebrate peptides displayed enhanced bactericidal activity against *E. coli*. Complete bacterial killing was observed at a concentration of 1 μM in human plasma, which is due to boosting complement activation, whereas higher concentrations of the peptides were required for their direct antimicrobial activity as seen from incubating the peptide in presence of buffer (Fig. 4). The growth of bacteria in buffer is slower than in citrated plasma, but the peptide-mediated killing in buffer is still less efficient than in citrated plasma.

### Effect of TFPI-2 derived peptides on blood coagulation/intrinsic pathway of coagulation

**Fig. 5:**
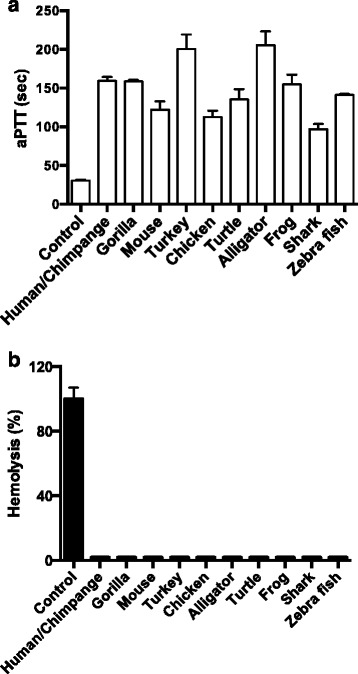
Anticoagulant effects of TFPI-2 derived peptides. **a** The activated partial thromboplastin time (aPTT) was determined by addition of buffer or 50 μM of different vertebrate TFPI-2 derived peptides to human plasma. Data are presented as clotting time in seconds; values are mean ± SD (*n* = 3). **b** Human erythrocytes were incubated with 60 μM of vertebrate TFPI-2 derived peptides. The hemoglobin release was measured at λ 540 nm and hemolysis was indicated in percentage, Triton X-100 treated sample being used a positive control

Previously, it was shown that the human TFPI-2 derived peptide EDC34 blocks the intrinsic pathway of coagulation in both human and murine plasma [22]. Based on these findings we investigated the anticoagulant activity of the vertebrate-derived TFPI-2 peptides by determining the prolongation of activated partial thromboplastin time (aPTT). As shown in Fig. 5a, all peptides effectively prolonged the normal clotting time on aPTT by more than 100 s when applied at a concentration of 50 μM. We further noted that all vertebrate-derived anticoagulant peptides tested are non-hemolytic at 60 μM and thus have potential to be used in therapeutic applications (Fig. 5b).

### Mouse C-terminal TFPI-2 derived VKG24 peptide provides protection against septic shock

**Fig. 6:**
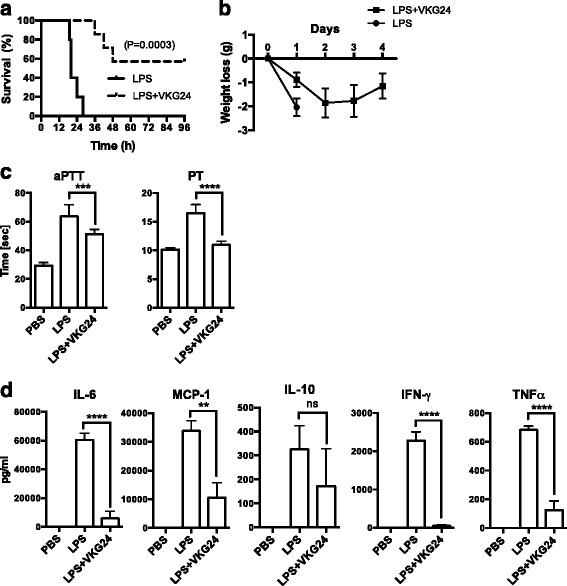
Treatment of LPS-induced septic shock mice with a mouse TFPI-2 derived peptide. Septic shock in Balb/c mice was induced by i.p. injection of 10 mg/kg *E. coli* LPS. Thirty minutes after LPS injection, VKG24 (0.5 mg/mouse or ~25 mg/kg body weight) or PBS was administrated i.p. **a** Mouse TFPI-2 derived peptide significantly increased survival of mice. Survival of mice was monitored for 7 days (only the first 96 h is shown in the figure). Statistical comparisons of survival curves were performed using the Mantel-Cox’s test. ***P ≤ 0.0003. **b** Weight was determined daily. **c** Clotting times of aPTT and PT was measured in citrate plasma 24 h after LPS injection (*n* = 8). **d** The indicated cytokines were analyzed in plasma 24 h after LPS injection (*n* = 8). Statistical analysis for C-D, one-way ANOVA with Tukey’s multiple comparisons post-test was used. **P ≤ 0.003, ****P ≤ 0.0001

Given the observed effects of the TFPI-2 C-terminal peptides, we made an attempt to find out whether these peptides are of therapeutic importance. To test whether the endogenous C-terminal TFPI-2 region is important in host defense, the mouse peptide VKG24 was chosen for a murine in vivo model. In vitro, VKG24 peptide displayed potent antimicrobial and anti-coagulant activities in mouse plasma (Additional file 2: Figure S2). Thus, the in vivo efficiency of VKG24 peptide was evaluated in a standardized mouse model of endotoxin-induced shock [22]. A dramatic improvement in the survival rate of the animals was seen after treatment with the peptide (~25 mg/kg body weight) (Fig. 6a). Peptide-treated animals started regaining their weight from day three (Fig. 6b). Previous studies have shown that thrombocytopenia is as an important indicator for the severity of sepsis and disseminated intravascular coagulation [23]. Therefore, activation of the intrinsic and extrinsic coagulation pathways was measured in citrate plasma of LPS-injected mice that received a VKG24 injection or were left untreated. The treatment showed significantly decreased coagulation aPTT and PT times in LPS challenged mice, indicating that the peptide reduced consumption of coagulation proteins (Fig. 6c). The levels were completely normalized in the survivors after seven days. Analyses of the cytokine profile 24 h after LPS injection showed significant reduction of IL-6, MCP-1, IFN-γ and IL-10, respectively (Fig. 6d). Thus, the results demonstrate that the mouse VKG24 peptide exerts potent immunomodulatory activity.

## Discussion

In the present study we aimed to study the host defence and immunomodulatory activities of the vertebrate C-terminal epitope of TFPI-2. Sequence analysis of this region in different vertebrate species showed that the exact amino acid sequence was not well conserved, whereas the net positive charge appeared to be preserved. These findings suggest an essential function of the charge. It can be speculated that there has been an evolutionary pressure to maintain the positive charge in the C-terminal region, as this is important in the host defence against bacterial pathogens. Within the last years, TFPI-2 has attracted increasing attention because of its ubiquitous presence, which leads to the deposition in a variety of tissues and particularly in the extracellular matrix. This likely reflects its potential as a central regulator of multiple biological processes involving the control of inflammatory reactions, matrix protease activity, coagulation, angiogenesis, and tumour growth. In previous studies we found that the proteolysis of human TFPI-2 generates C-terminal fragments, both in vivo and in vitro after digestion with neutrophil elastase. These findings are of importance in the context of proteolysis of TFPI-2 and possible release of bioactive host defence fragments [9, 10]. Notably, the observed generation of C-terminal fragments of TFPI-2 are very similar to that seen with TFPI-1, suggesting that the same cleavage sites is used by plasmin and thrombin to generate the C-terminal part of TFPI-1. Interestingly, previous data on TFPI-1 and TFPI-2 revealed that the release of C-terminal peptides may exert similar complement boosting effects [8–10]. It is also notable that the C-terminal peptide of TFPI-1, GGL27, and TFPI-2, EDC34 prolongs aPTT, thus further illustrating a functional overlap between the two TFPI proteins. In a broader perspective, a picture thus emerges; suggesting that C-terminal fragments from both TFPI-1 and TFPI-2 may be released during inflammation and infection, serving as modulators of both antimicrobial activity and coagulation. So far, available structural and functional data separate the C-terminal TFPI-2 peptides from the group of classical amphipathic antimicrobial peptides, such as the helical cathelicidin peptide LL-37 as well as defensins. Indeed, the EDC34 show a similarity to some linear peptides of low helical content, such as antimicrobial peptides derived from growth factors [24] as well as human kininogen, all displaying mostly random coil conformation in buffer and at lipid bilayers, the interactions dominated by electrostatic interactions [25].

It remains to be investigated whether TFPI-2 peptides may have similar actions as those reported here, although the absence of a marked boosting of activity in plasma suggests that complement interactions are characteristic for the TFPI-peptides. The detailed mode of action of these peptides remains to be explored, but it seems that they are involved in interactions with C1q and/or other molecules that can initiate the classical pathway of complement and lead to the formation of C3a or a MAC complex. Contact activation, involving degradation of human kininogen, and release of vasoactive bradykinin has been reported during various infective processes including sepsis [26, 27]. The finding that EDC34 inhibited contact activation in vitro is of importance, since a generalized activation of the system can be deleterious.

From a clinical perspective, many opportunistic bacteria can cause both localized and systemic infections and are responsible for considerable mortality or morbidity in patients suffering from burn wound infections, pneumonia, cystic fibrosis, intra-abdominal infections, chronic ulcers, and sepsis. Our results show a direct antimicrobial activity of the TFPI-2 peptide derived from different vertebrates species. The potent antimicrobial activity of these peptides was paired with boosting of complement activation and a modulation of the coagulation cascade. In vivo, VKG24 administration after LPS challenge protects mice and also significantly reduced aPTT and PT time. Therefore, our findings that VKG24 is able to significantly reduce LPS induced shock is particularly relevant, and implies a therapeutic potential for VKG24. The protective effect of mouse VKG24 in infections caused by Gram-negative pathogens may also apply to other vertebrate TFPI-2 C-terminal peptides.

## Conclusions

Several therapeutic strategies to combat severe infections including sepsis are based on supplementation of antibiotics with molecules exerting anti-coagulant and anti-inflammatory actions [28]. The dual effects of these natural peptides, for example the mouse derived VKG24 peptide, that can block contact activation and boost complement activation, thus represent, to our knowledge, a novel treatment concept, which could enable complement mediated bacterial clearance while inhibiting the deleterious effects of excessive procoagulant responses during infection with Gram-negative bacteria.

## Methods

### Peptides

Indicated peptides (Additional file 3: Table S1) were synthesized by Ontores (Shanghai, China). The purity (>98 %) of these peptides was confirmed by mass spectral analysis (MALDI-ToF Voyager).

### Microorganisms

The bacterial isolates *E. coli* ATCC 25922 and *P. aeruginosa* ATCC 27853 were obtained from the American Type Culture Collection.

### Radial diffusion assay (RDA)

Essentially as described earlier, bacteria were grown to mid-logarithmic phase in 10 ml of full-strength (3 % w/v) trypticase soy broth (TSB) (Becton-Dickinson, Cockeysville, MD). The microorganisms were then washed once with 10 mM Tris, pH 7.4. Subsequently, 4 × 10^6^ bacterial colony forming units (cfu) were added to 15 ml of the underlay agarose gel, consisting of 0.03 % (w/v) TSB, 1 % (w/v) low electroendosmosis type (EEO) agarose (Sigma, St Louis, MO) and 0.02 % (v/v) Tween 20 (Sigma). The underlay was poured into a Ø 144 mm petri dish. After agarose solidification, 4 mm-diameter wells were punched and 6 μl of test sample was added to each well. Plates were incubated at 37 °C for 3 h to allow diffusion of the peptides. The underlay gel was then covered with 15 ml of molten overlay (6 % TSB and 1 % Low-EEO agarose in distilled H_2_O). Antimicrobial activity of a peptide is visualized as a zone of clearing around each well after 18–24 h of incubation at 37 °C.

### Viable count analysis (VCA)

*E. coli* were grown to mid-exponential phase in Todd-Hewitt (TH). Bacteria were washed and diluted in 10 mM Tris, pH 7.4 either alone or with 20 % human or mouse (BALB/c) citrate-plasma. Bacterial (2 × 10^6^ cfu/ml) were incubated in 50 μl, at 37 °C for 1 h with the C-terminal TFPI-2 derived peptides at the indicated concentrations. Serial dilutions of the incubation mixture were plated on TH agar, followed by incubation at 37 °C overnight and cfu determination.

### LPS animal model

Male/female balb/c mice (8 weeks, 21 ± 5 g) were injected intraperitoneally (i.p.) with 10 mg *E. coli* 0111:B4 LPS (Sigma) per kg of body weight. Thirty minutes after LPS injection, 0.5 mg VKG24 (25 mg/kg) or PBS buffer alone (control) were injected i.p. into the mice. Status and weight were monitored daily for seven days. Mice where immobilization and/or shaking was observed were euthanized by an overdose of isoflurane (Abbott) and counted as non-survivors. For determination of cytokine levels in mouse plasma, animals were sacrificed 24 h after LPS injection. The blood was collected immediately by cardiac puncture into citrate tubes.

### Cytokine assay

Cytokines IL-6, IL-10, MCP-1, INF-γ and TNF-α were measured in plasma from mice infected by *E. coli* LPS (with or without peptide treatment) using the Cytometric bead array; mouse inflammation kit (Becton Dickinson AB) according to the manufacturer’s instructions. All plasma samples were stored at −80 °C before the analysis.

### Hemolysis assay

EDTA-blood was centrifuged at 800 × *g* for 10 min, where after plasma and buffy coat were removed. The erythrocytes were washed three times and resuspended in PBS, pH 7.4 to get a 5 % suspension. The cells were then incubated with end-over-end rotation for 60 min at 37 °C in the presence of peptides (60 μM). Triton X-100 (Sigma) 2 % served as positive control. The samples were then centrifuged at 800 × *g* for 10 min and the supernatant was transferred to a 96 well microtiter plate. The absorbance of hemoglobin release was measured at 540 nm and expressed as percentage of Triton X-100 induced hemolysis.

### Phylogenetic and sequence homology analyses

Various vertebrate TFPI-2 amino acid sequences, which are presently available, were retrieved from the NCBI, Ensembl and Uniprot. These sequences were aligned using Blosum 69 protein weight matrix settings using BioEdit software (Ibis Biosciences, Carlsbad, CA). Internal adjustments were made taking the structural alignment into account utilizing the ClustalW interface. Neighbor-joining method was used for phylogenetic tree construction, and the reliability of each branch was assessed using 1000 bootstrap replications using Mega-6.

### Clotting assays

All clotting times were analyzed using a coagulometer (Amelung, Lemgo, Germany). For determination of activated partial thromboplastin time (aPTT), 100 μl of a kaolin-containing solution (Technoclone) was added to the 100 μl of fresh citrate plasma or citrate plasma peptide mix and incubated at 37 °C for 200 s before clot formation was initiated by adding 100 μl of 30 mM fresh CaCl_2_ solution. For detection of prothrombin time (PT, thromboplastin reagent (Trinity Biotech)) 100 μl of fresh citrate plasma were incubated 60 s at 37 °C before clot formation was initiated by adding 100 μl of clotting reagent.

### Statistical analysis

Values are shown as mean with SEM. For statistical evaluation of two experimental groups, one-way with Tukey’s multiple comparisons post-test was used and for comparison of survival curves the Mantel-Cox’s test. Viable count and radial diffusion assay data are presented as mean with SD. All statistical evaluations were performed using the GraphPad Prism software 6.0 with *p- < 0.05, **p- < 0.01, *** < 0.001, ****p < 0.0001 and ns = not significant.

## Abbreviations

TFPI-2, tissue factor pathway inhibitor-2; aPTT, activated partial thromboplastin time; PT, prothrombin time

## Additional files

Additional file 1: Figure S1. Phylogenetic tree analysis of full-length and reminder protein except C-terminal TFPI-2 from vertebrates. Phylogenetic tree from 72 vertebrate TFPI-2 species was constructed using Neighbour-Joining tree with 1000 bootstrap replications on MEGA6. (PDF 54 kb) Additional file 2: Figure S2. Activities of TFPI-2 C-terminal derived peptides in mouse plasma. (Left) The bactericidal activity of mouse VKG24 peptide was assessed in physiological buffer conditions using viable count analysis. *E. coli* ATCC 25922 were grown to mid-logarithmic phase and incubated with varying concentrations of peptide. The antimicrobial activity was determined by plating serial dilutions of bacteria on TH agar plates and number of cfu was counted after overnight incubation. (Right) The activated partial thromboplastin time (aPTT) was determined by addition of buffer or 50 μM of VKG24 peptide to mouse plasma. Data are presented as clotting time in seconds; values are mean ± SD (*n* = 3). (PDF 32 kb) Additional file 3: Table S1. Selected vertebrate TFPI-2 C-terminal peptides showing sequence entry name, species, sequence, charge and hydrophobicity. (PDF 73 kb)
