# Supplementary figures and images for: Vertebrate TFPI-2 C-terminal peptides exert therapeutic applications against Gram-negative infections
Source: BMC Microbiol. 2016 Jun 27;16:129. doi: 10.1186/s12866-016-0750-3 (PMC4924314; doi:10.1186/s12866-016-0750-3)

Full length protein

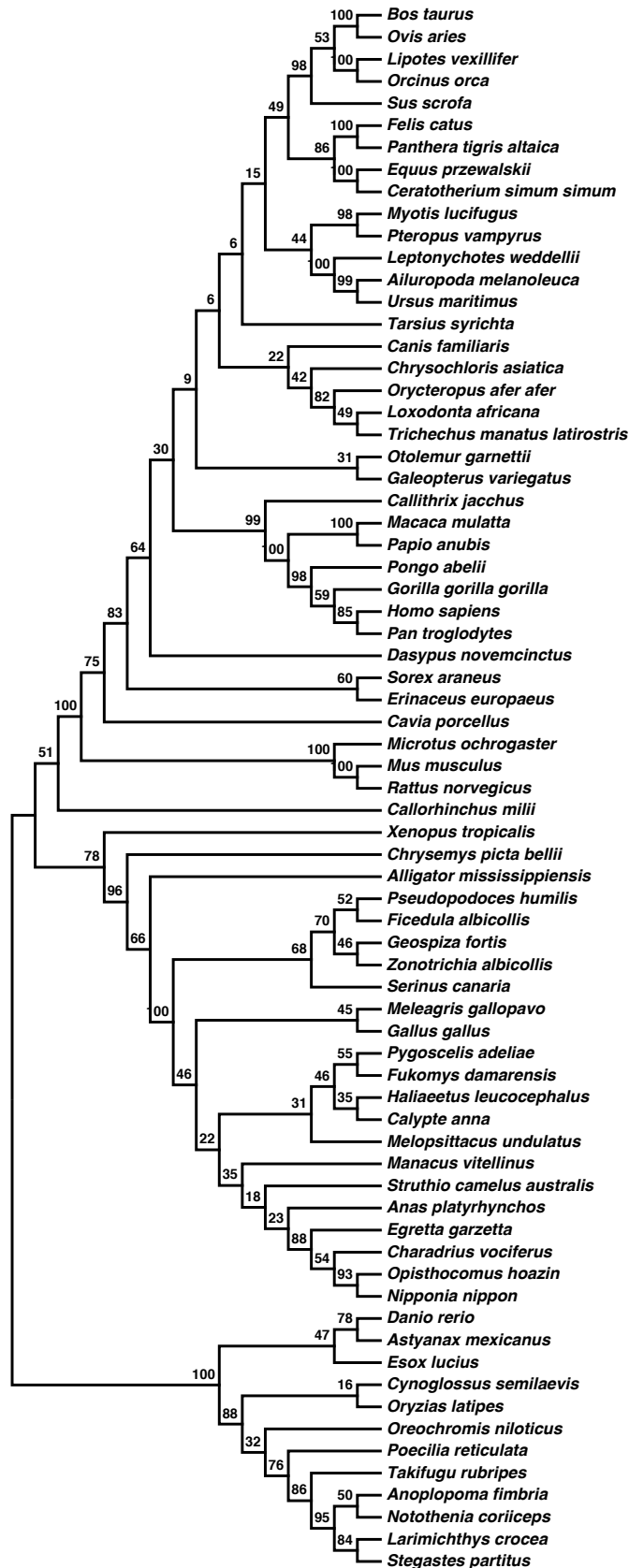

Remainder protein except C-terminus

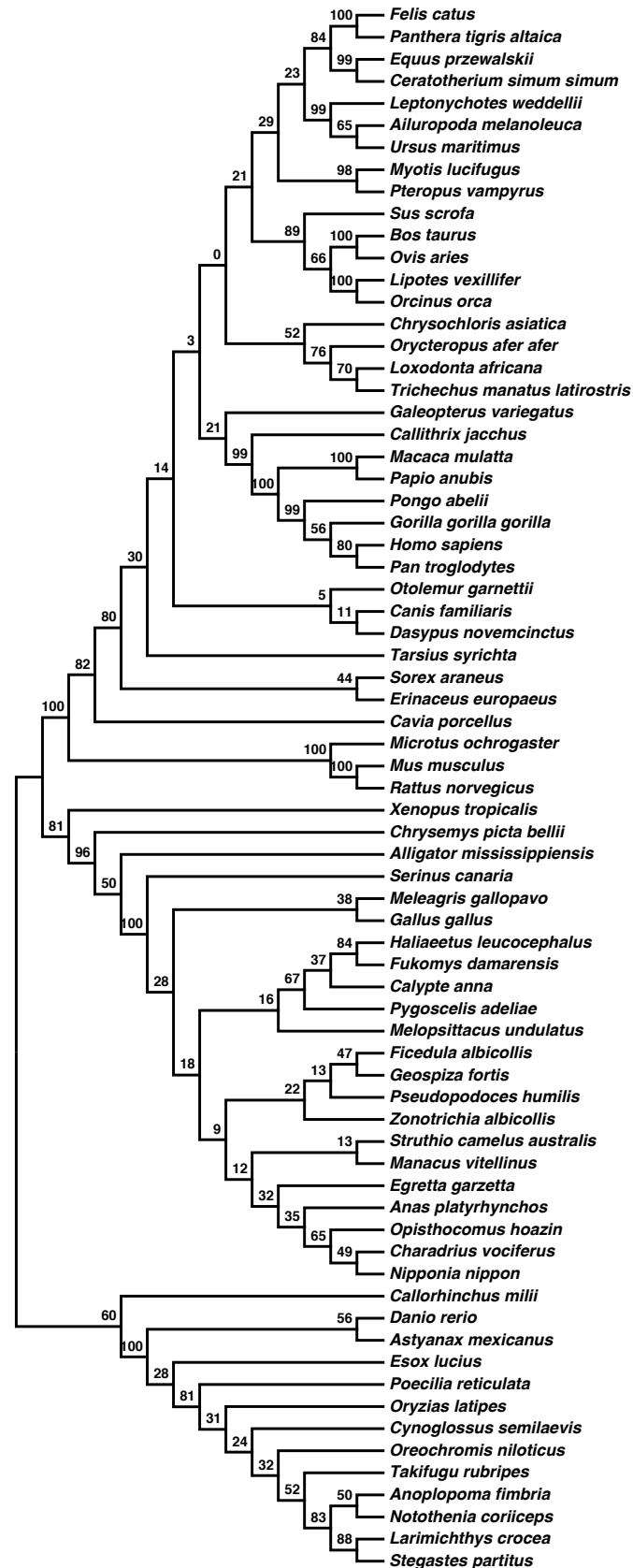

Supplement: Additional file 1: Figure S1. — Phylogenetic tree analysis of full-length and reminder protein except C-terminal TFPI-2 from vertebrates. Phylogenetic tree from 72 vertebrate TFPI-2 species was constructed using Neighbour-Joining tree with 1000 bootstrap replications on MEGA6. (PDF 54 kb) [file 12866_2016_750_MOESM1_ESM.pdf]

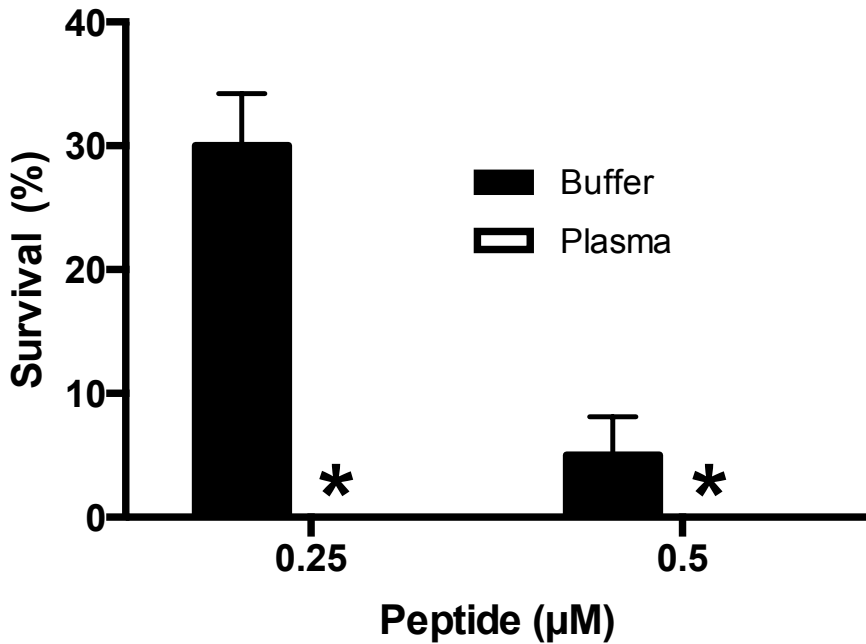

Supplement: Additional file 2: Figure S2. — Activities of TFPI-2 C-terminal derived peptides in mouse plasma. (Left) The bactericidal activity of mouse VKG24 peptide was assessed in physiological buffer conditions using viable count analysis. E. coli ATCC 25922 were grown to mid-logarithmic phase and incubated with varying concentrations of peptide. The antimicrobial activity was determined by plating serial dilutions of bacteria on TH agar plates and number of cfu was counted after overnight incubation. (Right) The activated partial thromboplastin time (aPTT) was determined by addition of buffer or 50 μM of VKG24 peptide to mouse plasma. Data are presented as clotting time in seconds; values are mean ± SD (n = 3). (PDF 32 kb) [file 12866_2016_750_MOESM2_ESM.pdf]
