# Supplementary material for: Vertebrate TFPI-2 C-terminal peptides exert therapeutic applications against Gram-negative infections
Source: BMC Microbiol. 2016 Jun 27;16:129. doi: 10.1186/s12866-016-0750-3 (PMC4924314; doi:10.1186/s12866-016-0750-3)
Supplement: Additional file 3: Table S1. — Selected vertebrate TFPI-2 C-terminal peptides showing sequence entry name, species, sequence, charge and hydrophobicity. (PDF 73 kb) [file 12866_2016_750_MOESM3_ESM.pdf]

Entry name	Peptide code	Species comon name	Sequence	Charge	Hydrophobicity
P48307/H2QUX8	AKA27	Human/chimpange	AKALKKKKMPKLRFASRIRKIRKKQF	14	0.204
O35536	VKG24	Mouse	VKGWKKPKRWKIGDFLPRFWKHLS	7	0.274
XP_010710984	KKG27	Turkey	KKGSQKPTISKPRNLLRRKMMRKLIKK	12	0.09
XP_418662.2	RKG27	Chicken	RKGSQKPTINKSRSLLRRKMMRKLIKK	12	0.105
Q1WCN6	GSK27	Zebrafish	GSKRWSPTKKSVRVSKQYLRRVKPQPS	9	0.27
XP_005308608.1	KKA27	Turtle	KKAGSKKSSFKKSRNKLPKTMRKLPEK	11	0.393
XP_006258519.1	RKA27	Alligator	RKAGNKKPRIKPKSKVMRKMMRKLQKN	13	0.303
XP_007903512.1	QKR27	Shark	QKRNKKKTSQKYPTASITRKVVKKNSR	11	0.116
Q5FVY6	KKG20	Frog	KKGSKRPRNRNRIRVPRIQS	9	0.152
G3QPC8	AKA27	Gorilla	AKALKRKKKMPKLRFASRIRKIRKKQF	14	0.205
